# Stool Gluten Peptide Detection Is Superior to Urinary Analysis, Coeliac Serology, Dietary Adherence Scores and Symptoms in the Detection of Intermittent Gluten Exposure in Coeliac Disease: A Randomised, Placebo-Controlled, Low-Dose Gluten Challenge Study

**DOI:** 10.3390/nu16020279

**Published:** 2024-01-17

**Authors:** Amy K. Russell, Erin C. Lucas, Lee M. Henneken, Catherine J. Pizzey, Dean Clarke, Anna Myleus, Jason A. Tye-Din

**Affiliations:** 1 Immunology Division, The Walter and Eliza Hall Institute, Parkville, VIC 3052, Australia; russell.a@wehi.edu.au (A.K.R.);; 2 Department of Gastroenterology, The Royal Melbourne Hospital, Parkville, VIC 3052, Australia; 3 National Measurement Institute, Port Melbourne, VIC 3207, Australia; 4 Department of Public Health and Clinical Medicine, Family Medicine, Umea University, 901 87 Umea, Sweden; 5 Department of Medical Biology, University of Melbourne, Parkville, VIC 3052, Australia; 6 The Murdoch Children’s Research Institute, Parkville, VIC 3052, Australia

**Keywords:** coeliac disease, gluten immunogenic peptides, gluten excretion stool, gluten-free diet monitoring

## Abstract

Monitoring adherence to a gluten-free diet is an important goal of coeliac disease management. Urine and stool gluten immunogenic peptide (GIP) assays provide an objective readout of gluten ingestion, with the former favoured due to its convenience and acceptability. This study assessed stool GIP excretion after low-dose gluten challenge designed to mimic accidental gluten exposure. A total of 52 coeliac participants undertook a randomised, double-blind gluten (50–1000 mg) or placebo challenge. Stool and urinary GIP, serology, dietary adherence and symptoms were assessed. Stool GIP was 100% sensitive for gluten intake ≥250 mg and 71% for 50 mg. Peak GIP detection was 12–36 h after gluten exposure. The mean stool GIP after 1000 mg gluten ingestion remained above the limit of quantification for 5 days. Urine GIP assessment had poor sensitivity for GIP excretion compared to stool. Serology, dietary adherence score and symptoms did not correlate with gluten excretion during lead-in. We conclude that stool GIP detection is highly sensitive, with levels related to gluten dose and time from ingestion. Weekly or bi-weekly testing will detect low-level exposure more effectively than urine GIP assessments or traditional methods. In this seronegative, apparently well-treated cohort, a high frequency of baseline-positive GIP suggests ongoing gluten exposure, but the assessment of patient behaviour and assay specificity is needed.

## 1. Introduction

Coeliac disease is a prevalent immune-mediated enteropathy precipitated by exposure to dietary gluten [1]. Chronic gluten exposure is associated with enteropathy, positive coeliac serology, adverse symptoms and an increased risk of complications such as osteoporosis and lymphoproliferative malignancy [2]. A strict gluten-free diet to remove the precipitating dietary factor that causes these effects supports mucosal healing, the resolution of symptoms, improved quality of life and the reduced risk of complications. However, a major challenge is maintaining strict dietary adherence, as this treatment is burdensome and restrictive [3,4]. Gluten cross-contamination is a common contributor to inadvertent gluten intake [5,6]. As a result, the most common cause of persistent symptoms and enteropathy in treated patients is ongoing gluten exposure [7].

Given the importance of adequate dietary gluten exclusion, monitoring patient adherence to a gluten-free diet is a central tenet of coeliac disease management; however, accurate assessment is difficult. Indirect measures of adherence are frequently employed and include dietary assessment, dietary-adherence scores, the evaluation of clinical status and coeliac serology [2]. Unfortunately, these lack sensitivity to detect gluten exposure and correlate poorly with gluten intake and mucosal healing [8,9,10]. While small intestinal histology informs on coeliac disease activity, when enteropathy is detected, it is not possible to determine if these changes are due to gluten intake (and if so, how much) or caused by refractory coeliac disease, which by definition occurs in the absence of gluten intake. Further, relying on histology obtained via invasive gastroscopy is impractical for regular monitoring.

The gluten immunogenic peptide (GIP) assay, which detects a major wheat gliadin peptide in urine or stool (G12 antibody), can objectively and sensitively measure the presence of gluten in stool or urine [11,12,13]. Based on high excreted GIP signals in treated coeliac disease, this tool has highlighted that inadvertent gluten exposure in treated coeliac disease appears to be more frequent than previously realised [14,15,16,17].

Establishing GIP testing in the clinic requires an understanding of the link between the measured GIP signal and the amount of gluten ingested as well as determining if stool or urine is the most informative medium to sample. Unfortunately, studies to date are mostly observational in design and cannot inform on the relationship between gluten intake and GIP signal as the amount of gluten ingested is unable to be determined. Further, there is a bias towards employing urine GIP assessment over stool, as reflected by the predominance of the published literature utilising this approach, as urine is easier to collect and more acceptable to patients. The clinical utility of urine testing compared to stool has not been determined. While small controlled gluten challenge studies in healthy volunteers (*n =* 20) [18] and coeliac patients (*n* = 15) has shown the high sensitivity of urine GIP assessment [19], no studies to assess stool GIP after controlled gluten challenge in coeliac patients have been reported to enable direct comparisons.

Given these shortcomings, we undertook a randomised, double-blind, placebo-controlled gluten challenge study to assess the stool excretion dynamics of controlled gluten exposure in patients with coeliac disease and the relationship between gluten intake, GIP signal, coeliac serology and symptoms. To support clinical relevance and implementation, we assessed low levels of gluten to simulate the “real-life” scenario where coeliac patients attempting to adhere to a gluten-free diet are intermittently exposed to low levels of gluten.

## 2. Materials and Methods

### 2.1. Overall Study Design

**Figure 1:**
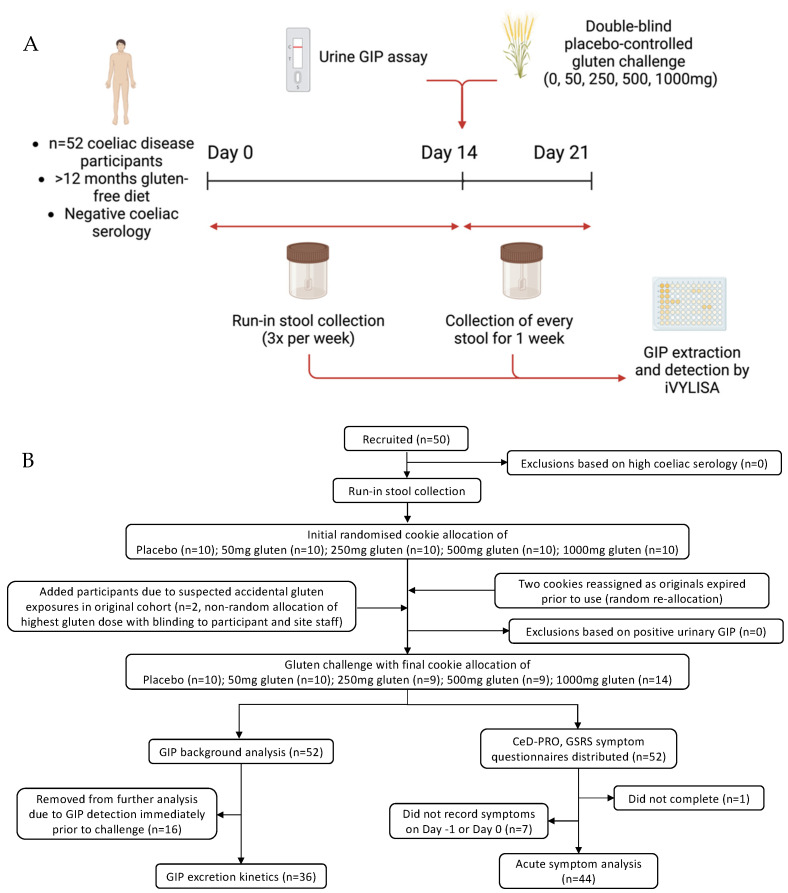
Outline showing (**A**) study schematic and (**B**) enrolment flow chart.

We performed a randomised, double-blind, low-dose, placebo-controlled gluten challenge study (Figure 1) in adults with treated coeliac disease conducted through an academic centre (Walter and Eliza Hall Institute) and clinical site (Royal Melbourne Hospital). The recruitment sample size was ultimately shaped by practical constraints, and formal powering calculations were not performed. Participants were following a gluten-free diet for at least 12 months and had negative coeliac serology at baseline. Following a two-week run-in period, participants who had a negative urinary GIP went on to consume a single gluten-free cookie spiked with one of four randomly allocated gluten doses or a placebo. All stool samples were assessed for GIP content. Symptom data, dietary adherence (daily) and coeliac serology (baseline and end of study) were also assessed.

### 2.2. Ethics Statement

The study was conducted according to the guidelines of the Declaration of Helsinki and approved by the Ethics Committee of Melbourne Health and the Walter and Eliza Hall Institute (protocol codes 2020.162 and 20/21, respectively, and dates of approval 13 November 2020 and 5 January 2021, respectively). Written informed consent was obtained from all participants. Australian New Zealand Clinical Trials Registry number: ACTRN12620000259943.

### 2.3. Participants

Participants were enrolled from February 2020 to April 2021. Inclusion criteria were (i) adults aged between 18 and 70 years with a medically confirmed diagnosis of coeliac disease, (ii) adherence to a gluten-free diet for at least 12 months, (iii) negative transglutaminase-IgA and deamidated gliadin peptide-IgG at baseline and (iv) a willingness to undertake regular stool collection and potential gluten ingestion. Coeliac disease diagnosis was based on documented evidence of prior duodenal villous atrophy (Marsh 3) associated with positive transglutaminase-IgA and/or deamidated gliadin peptide-IgG and a supportive clinical history. Exclusion criteria included poorly controlled coeliac disease based on frequent, persistent symptoms, a diagnosis of non-responsive coeliac disease or refractory coeliac disease and non-adherence to the gluten-free diet. The Coeliac Dietary Adherence Test (CDAT) was administered pre- and post-study [20]. Current and presenting symptoms, medications and general health information was recorded.

### 2.4. Run-in Period

In the two-week run-in period, participants followed their gluten-free diet with stool collected three times per week for stool GIP assessment. Participants reported any suspected episodes of gluten intake, and if these were recorded, an additional washout period of two weeks was observed before reassessing eligibility.

### 2.5. Gluten or Placebo Cookies

Gluten-free cookies (Gluten Free Chocolate chip cookie, Betty Crocker, Golden Valley, MN, USA) were prepared as per the manufacturer’s instructions by a dedicated, unblinded trial co-ordinator not involved in participant interactions. The packet cookie mixture and rice flour (McKenzies, Altona, VIC, Australia) used for the placebo were tested using R5 ELISA (R-Biopharm, Darmstadt, Germany) to confirm they contained no detectable gluten (National Measurement Institute, NMI, Port Melbourne, VIC, Australia). Immediately prior to baking, vital wheat gluten (Bob’s Red Mill, Milwaukie, WI, USA) confirmed to be low in FODMAP content (Monash University) or gluten-free rice flour was added to each individual cookie dough. Each set of cookies with a specific dose level was prepared as a discrete batch, with the kitchen and preparation utensils cleaned thoroughly in between. The amount of vital wheat gluten added was based on the calculated gluten content of 63.04 g per 100 g, so the final estimated gluten content of each cookie was 50 mg, 250 mg, 500 mg or 1000 mg. Then, 1000 mg of rice flour was added to the placebo cookies. Following baking, a spare cookie from the 1000 mg dose batch was tested by RIDASCREEN Total Gluten ELISA (R-Biopharm) which confirmed the presence of 975 mg gluten (NMI). The cookies containing gluten and rice flour had an indistinguishable taste and texture.

### 2.6. Cookie Randomisation

Participants were randomly assigned into the active or placebo arms in a 1:1:1:1:1 ratio in randomly selected block sizes of five using the GraphPad Randomization Calculator. Multiple randomisation lists were generated, each containing all five doses in a random order. Participants were sequentially assigned to doses based on the randomisation lists. Two events led to an uneven sample size in cohorts: Firstly, two people who delayed their start were reallocated fresh cookies with a new randomisation to replace the original expired cookies. Secondly, two people reported suspected gluten exposure prior to cookie challenge; therefore, two additional participants were added by an investigator and allocated the 1000 mg dose. This investigator did not interact with participants or trial staff to maintain study blinding. Study site personnel received the unique patient randomisation number, date of randomisation and assignment but remained blinded to the identity of the assignment until the database was unlocked and the study unblinded.

### 2.7. Cookie Ingestion

On the day of the cookie challenge, participants were instructed to perform a self-test for recent gluten exposure on the first urination of the day (GlutenDetect urine home-use kit, Biomedal S.L., Seville, Spain). If positive, participants were instructed not to proceed with the challenge. Participants consumed their allocated cookie in the morning after an overnight fast and were instructed to maintain a strict gluten-free diet at all other times. For analysis, days post-cookie ingestion were broken into 24 h blocks starting from 12 h after the challenge, i.e., day of challenge = 0–12 h post-challenge, Day 1 = 13–36 h post-challenge and so on up to Day 7 post-challenge. The 12 h interval was selected as the cookies were consumed in the morning and stools were rarely passed overnight.

### 2.8. Stool Collection

Participants were instructed to collect stool samples three times per week during the two-week run-in and every movement passed after the cookie challenge for a minimum of one week. Time-labelled samples were placed into a clip-lock container and stored in a domestic freezer until transport to the WEHI laboratory.

### 2.9. Symptom Record

The Gastrointestinal Symptom Rating Scale (GSRS) [21] was completed at baseline (prior to run-in period) and repeated at the end of the study, and a modified Coeliac Disease Patient Reported Outcome measure (CeD-PRO) [22] was completed daily throughout the study. A diary was used by participants to record when a bowel movement was passed and if inadvertent gluten ingestion was suspected. Participants were asked if they thought the cookie they consumed contained gluten.

### 2.10. Quantification of GIP in Stool

All stool samples for each participant were extracted and analysed together using the iVYLISA GIP stool test (Biomedal S.L., Seville, Spain) according to kit instructions with minor additions that included thorough manual homogenisation and the removal of solid food matter. After processing, extracts were stored at −20 °C for up to two weeks before analysis. iVYLISA results were expressed as μg of GIP/gram stool.

### 2.11. Analytical Performance

The intra-assay CV of the iVYLISA GIP stool test was determined from 6 different stool samples run with 6 replicates with a mean CV of 5.6%. The inter-assay CV, as measured by kit inner-control in 25 runs, was 3.8%. The inter-assay CV determined using 13 stool samples run on 2 different days was 31.9% (49.0% for samples below 0.156 μg/gram stool, compared to 17.3% for samples above this). This variability at lower GIP concentrations has been reported previously by others [23], where samples below 0.156 μg/gram were considered to be below the limit of quantification (LOQ) due to inconsistent results. Therefore, we elected to use 0.156 μg/gram stool as the LOQ in our analyses, and results below this were classified as GIP negative.

### 2.12. Statistical Analyses

Summary statistics, medians and IQRs, were presented for continuous data, and frequencies and percentages were presented for categorical data. Wilcoxon signed rank tests were used to compare coeliac serology and symptom assessments pre- and post-challenge. Paired *t* tests were used to compare changes in GIP excretion before and after cookie challenge. Spearman analysis was used to correlate GIP excretion levels and symptoms. The Kruskal–Wallis test was used to determine the relationship between ingested gluten dose and GIP excretion. All statistical analyses were performed using GraphPad Prism version 9.

## 3. Results

### 3.1. Participant Characteristics

The flow of participants is shown in Figure 1. In total, 52 coeliac disease patients (36 (69%) female, median age 55 years) completed the study (Table 1). One participant reported suspected gluten exposure during the run-in, and a 2-week washout period was applied. All participants reported negative urine GIP immediately prior to cookie challenge. Stool collection ranged between three and eight samples during the run-in period. The baseline CDAT completed by 51/52 participants suggested “excellent” adherence (CDAT 7; *n* = 2; 3.9%), “very good” (CDAT 8–12; *n* = 35; 68.6%) and “insufficient” (CDAT 13–17; *n* = 14; 27.5%). None scored “poor” (CDAT > 17). Of the 14 that scored “insufficient”, 12 reported never deliberately consuming gluten and 2 reported the infrequent intake of small amounts, e.g., soy sauce less than once every 1 month or 12 months, respectively.

### 3.2. Baseline Stool and Urine GIP

**Figure 2:**
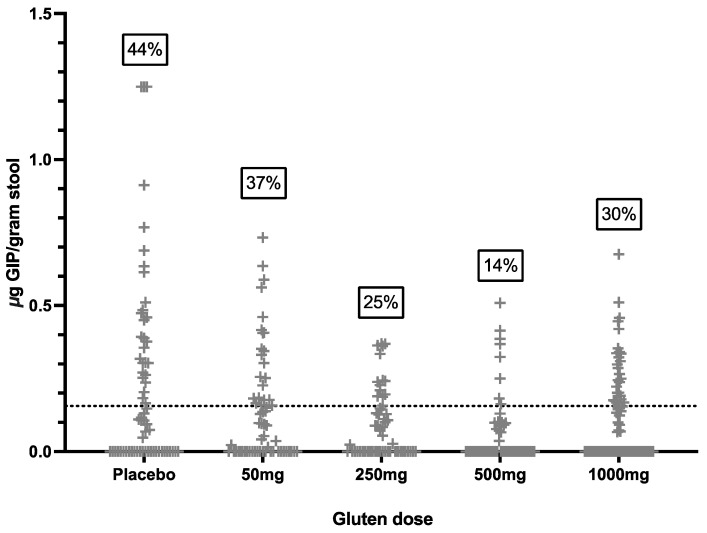
Concentration of stool GIP in all samples collected prior to the cookie challenge. Dotted line represents assay LOQ cut-off of 0.156 μg/gram stool. Numbers in boxes indicate percent of samples positive for GIP, i.e., above cut-off.

A total of 321 stool samples were collected prior to cookie consumption, and 98 were positive for GIP (31%). In total, 40/52 (77%) participants had at least one stool sample positive for GIP pre-cookie (Figure 2), disconcordant with the 52/52 (100%) negative urine GIP assays immediately prior to the cookie. The findings suggest a high rate of background gluten ingestion despite negative coeliac serology, most reporting good dietary adherence and negative urine GIP.

### 3.3. Stool GIP after Cookie Ingestion

**Figure 3:**
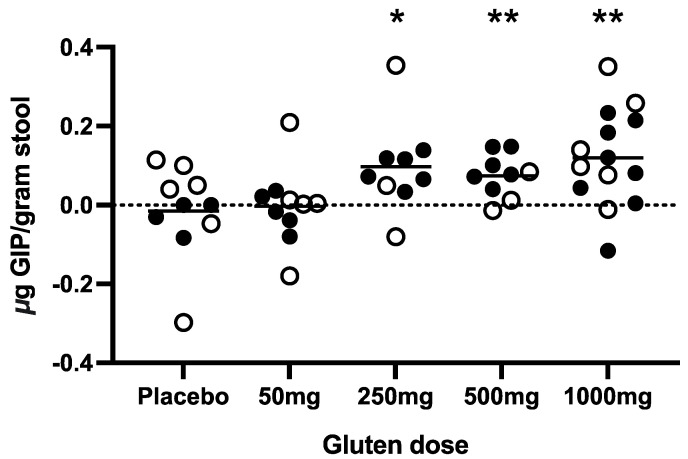
Changes in stool GIP post-challenge were assessed for each cohort (total *n* = 52). Each point represents the participant’s change in mean GIP pre-challenge to mean GIP post-challenge. Hollow circles indicate participants excluded from further analysis due to GIP detection in the stool sample immediately before the cookie. Short lines represent means for each dose cohort. Statistical differences were determined using paired two-tailed *t*-tests for values pre- vs. post-challenge. * *p* < 0.05, ** *p* < 0.01.

There was a statistically significant increase in the mean daily stool GIP level post-cookie (days 0–8) compared to pre-cookie (Days −22 to −1) in the 250 mg, 500 mg and 1000 mg gluten cohorts (Figure 3). To better define stool GIP excretion independent of high background, all participants with a positive GIP signal in the sample collected immediately prior to the cookie were excluded from the analysis (revised cohort for analysis: placebo *n* = 4; gluten 50 mg *n* = 7; 250 mg *n* = 8; 500 mg *n* = 7; and 1000 mg *n* = 10).

This generated a clearer relationship between the cookie gluten dose and stool GIP excretion (the mean peak stool GIP and area under the receiver operating characteristic curve (AUC) of stool GIP; Supplementary Figure S1), with gluten dose and time from ingestion being the two major variables impacting the stool GIP signal. There was a significantly higher peak stool GIP after 1000 mg gluten exposure compared to the placebo alone (*p* = 0.002), and the AUCs of the 250 mg (*p* = 0.018) and 1000 mg (*p* = 0.009) doses were significantly higher than the placebo alone (Kruskal–Wallis test). There was no correlation (ANOVA) between bowel movement frequency and time of peak GIP (*p* = 0.913) or timing of first GIP detection (*p* = 0.289). Gluten exposure did not significantly affect stool frequency, as measured by comparing the average number of movements per day before (1.4) and after (1.5) challenge (paired *t*-test; *p* = 0.128).

**Figure 4:**
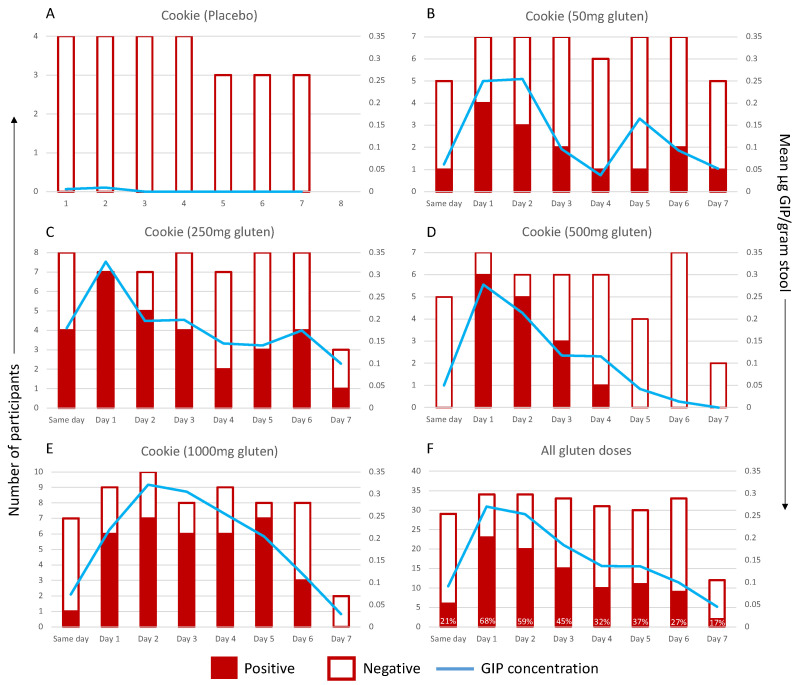
GIP excretion kinetics following known dose of: (**A**) placebo, (**B**) 50 mg of gluten, (**C**) 250 mg of gluten, (**D**) 500 mg of gluten or (**E**) 1000 mg of gluten. GIP excretion after any gluten dose (50–1000 mg) is shown in (**F**), with values showing percentage of samples positive for GIP in that time frame. Positive (red) and negative (white) samples by day per participant are graphed on the primary (left) axis. The mean GIP concentration is represented by the blue line and the secondary (right) axis. The *x*-axis shows time after cookie ingestion: Same day, 0–12 h; Day 1, 13–26 h; Day 2, 37–60 h; Day 3, 61–84 h; Day 4, 85–108 h; Day 5, 109–132 h; Day 6, 133–156 h; Day 7, 157–180 h.

The data were then represented in a binary fashion, where positive GIP samples represented a GIP concentration above the LOQ (0.156 μg GIP/gram stool) and negative samples for GIP were below the LOQ (Figure 4A–E). If multiple stool samples were collected in one 24 h period, the mean GIP concentration was calculated. A stool GIP above the LOQ was not detected after the placebo but was detectable in five out of seven participants consuming a cookie with 50 mg of gluten and in every participant consuming the cookies with 250–1000 mg of gluten. A higher gluten dose led to more prolonged detection, with the mean GIP remaining above the LOQ for 5 days after 1000 mg of gluten, compared to 2 days following 50 mg. For each dose, the mean peak GIP concentration occurred 1–2 days post-challenge. The timing of peaks varied between patients and cohorts: 50 mg cohort: 24–54 h post-cookie; 250 mg: 0–74 h; 500 mg: 20–53 h; and 1000 mg: 25–130 h.

### 3.4. Symptoms after Cookie Challenge

**Figure 5:**
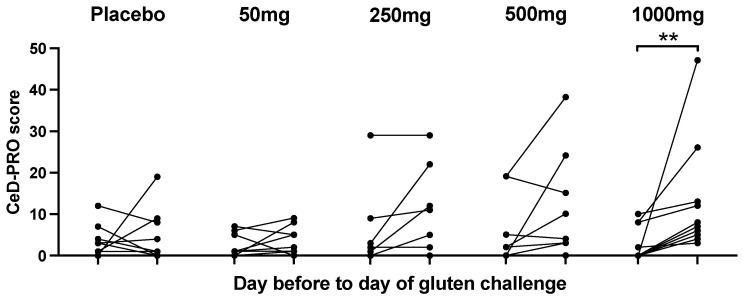
Acute total CeD-PRO symptom changes pre- and post-gluten cookie challenge. ** *p* < 0.05; Wilcoxon matched pairs test.

Symptoms were recorded during the two-week run-in period and after cookie ingestion by all participants except for two (4%) who remained symptom-free throughout the entire study. The gluten cookie was well tolerated. The mean daily CeD-PRO symptom score was low, indicating mild symptoms, with the most common being tiredness, gas and bloating, although these also occurred in the placebo cohort. Vomiting was observed after the 500 mg or 1000 mg gluten dose in one and three participants, respectively (Supplementary Figure S2). The CeD-PRO symptom score on day −1 was compared to the CeD-PRO symptom score on day 0 (Figure 5). There was a statistically significant change in total symptoms after 1000 mg of gluten (*p* = 0.002, Wilcoxon test), and compared to the placebo, there was more frequent and severe nausea, vomiting, abdominal cramping or pain, bloating, constipation and tiredness. Total GSRS scores and CD-GSRS scores showed no statistically significant change after gluten challenge at any dose.

### 3.5. Correlation of Gluten Intake with Symptoms, Dietary Adherence Score and Coeliac Serology

There was no correlation between post-cookie stool GIP AUC or peak GIP with total CeD-PRO symptom score (AUC rho = −0.079, *p* = 0.580 and peak rho = −0.145, *p* = 0.308, Spearman rank-order correlation). On direct questioning, few participants correctly identified when the cookie they consumed contained gluten. Ranked stool GIP levels prior to challenge showed no significant difference in CDAT score (*p* = 0.856, Kruskal–Wallis) (Supplementary Figure S3). Transglutaminase-IgA and deamidated gluten peptide-IgG remained negative at the end of the study in all participants.

## 4. Discussion

The role of urine or stool GIP assessment to inform the clinical care of coeliac disease is supported by the assay’s high sensitivity, but uncertainty about how it is applied in the clinic has limited its widespread adoption. To our knowledge, this is the first controlled gluten challenge study undertaken in people with coeliac disease to examine gluten excretion in stool following gluten ingestion at doses simulating those caused by inadvertent exposure, which is generally regarded in the order of 1000–2000 mg or less. By describing the excretion dynamics of gluten in stool using GIP assessment in coeliac patients, our findings highlight the value of stool over urine GIP testing to detect low-level, intermittent exposure and further underscore the limited value of utilising symptoms, patients’ self-report of gluten intake, coeliac serology and the CDAT dietary adherence score as markers of gluten exposure.

The optimal number and timing of GIP testing has not been clearly answered by prior observational studies [24]. We showed that across all doses of gluten studied, GIP assessment will detect 68% of ingestions the day after exposure to an unknown dose of gluten. Weekly stool testing should detect most episodes of higher level gluten ingestion (1000 mg or more) occurring within the preceding week and also low-level exposure within the prior 36 h. Separate to gluten dose, it is likely that patient-specific factors such as gastric emptying and intestinal transit times and the effects of microbial breakdown could influence excretion kinetics; however, we showed no association between more frequent bowel movements and timing to peak GIP or first GIP detection. However, as participants did not report significant diarrhoea or constipation in this study, we cannot exclude an effect of these on stool GIP sensitivity. For patients with coeliac disease attempting a strict gluten-free diet, the commonest real-world scenario is that of unintended low-level and intermittent gluten exposure, which is essentially an unpredictable event that patients may not be aware of. Based on our findings, multiple stool GIP assessments performed on a weekly or bi-weekly basis are likely to provide optimal sensitivity for detecting gluten exposure. The clinical significance of infrequent episodes of gluten intake not detected by this approach remains unclear, and given the wide variability in immune and histologic sensitivity to gluten in coeliac disease, is likely to differ between coeliac patients.

The field has significantly greater enthusiasm for applying the urine GIP assay over that of stool, largely because urine is more convenient and acceptable for patients to collect. However, we showed urine GIP testing failed to detect a single instance of unintended gluten exposure compared to 31% of single time points being identified by stool GIP testing. Of note, this was based on the lateral flow home-kit assay administered by patients and not the urine GIP ELISA assay which is likely to be more sensitive; we also asked patients to test their early morning urine which, through dilutional effects, could lead to a lower GIP signal. While it reasonable to assume the urinary assay was false negative and the stool GIP assay was true positive, we cannot discount the possibility that the urine assay was true negative and the stool assay was false positive. Nevertheless, our demonstration of the kinetics of stool GIP excretion confirm it is more sensitive for the detection of intermittent gluten exposure than urine, where urinary GIP levels peak substantially more acutely between 4.5 and 16 h after gluten ingestion [19]. Thus, stool GIP assessment would be of particular value in the real-world scenario of low-level and/or intermittent gluten intake. This would make it ideally suited to assess coeliac patients with persistent symptoms and enteropathy despite treatment (non-responsive coeliac disease) and in differentiating active disease due to gluten exposure from refractory coeliac disease, which, by definition, occurs in the absence of gluten intake [7].

Stool and urine GIP studies consistently reveal high GIP detection rates in treated coeliac disease, with higher detection associated with more regular testing. Positive urine or stool GIP have been detected in 25–48% of treated coeliac patients based on one or two collected samples [13,23,25] to 69% (twice-weekly stool GIPs performed four times over two years) [16] and up to 89% when samples were collected three times per week for four weeks [17]. We showed 77% of treated coeliac patients had at least one positive stool GIP, but this is reduced to 16/52 (31%) if only a single stool was assessed prior to the cookie. Our findings are consistent with these prior studies, which have all been interpreted to suggest that even in motivated coeliac patients attempting a strict gluten-free diet, it remains aspirational as opposed to readily achieved [14]. However, we argue there remains a clinically important need to examine the basis for high GIP signals in treated coeliac disease, including the role of patient behaviour and dietary choices and the specificity of the GIP assay. While studies have confirmed the high accuracy of the G12 antibody for gluten when employed as a food test [26,27], the complex nature of stool, including the fact that it contains enzymatically digested proteins, raises the possibility that the G12 antibody may perform differently in this matrix compared to food, and further assessment is needed.

A strength of this study is the use of a double-blind, placebo-controlled design and purified gluten confirmed to be low in fermentable carbohydrates (FODMAPs). This controlled for nocebo effects and non-specific functional gut symptoms triggered by FODMAPs [28,29]. Several participants in the placebo cohort reported an increase in symptoms after the challenge, consistent with a nocebo effect, supporting the value of double-blinding. The low-dose challenge was well tolerated, and whilst there was an increase in acute symptoms following the 1000 mg gluten dose, adverse symptoms were short-lived and resolved fully. The high rate of background symptoms in the placebo cohort suggests that true gluten-induced symptomatic episodes may be hard to detect, and this is supported by previous studies [30]. As symptoms prior to cookie ingestion did not correlate with stool GIP levels, we speculate that at least some were unrelated to gluten and caused by other conditions such as irritable bowel syndrome, which is a common comorbidity in coeliac disease [31,32]. There is enthusiasm for the use of GIP assessment in clinical trials, with urine GIP assessment recently used to inform on the efficacy of a gluten-degrading therapy for coeliac disease [33]. Some drug developers aim to utilise the assay to monitor gluten exposure during clinical studies. By informing on GIP and symptom effects of low-level gluten intake, our findings have important implications for the design and interpretation of gluten exposure studies in drug development trials designed to simulate real-life intermittent gluten exposure.

There are several limitations to this study. Even though this is the largest controlled study to assess stool GIP excretion in coeliac disease, after accounting for background noise, the final numbers in each dose cohort were relatively small. However, the consistent dose–response findings across all cohorts support clear conclusions about excretion kinetics. We attempted to minimise background gluten exposure by the use of the screening urinary GIP assay, but this demonstrated poor concordance with stool GIP, suggesting low sensitivity, which was an unforeseen event. A methodological consideration is that we relied on participants’ collection of each sample from several portions of the stool, which could introduce sampling inconsistencies. As it was not feasible to collect the entire stool or homogenise it, we mitigated this issue by frequent stool sampling, and our high GIP detection rate suggests our approach did not impair sensitivity. Finally, our study did not correlate stool GIP with small intestinal histology. This is because our focus was on the relationship between controlled gluten intake and its excretion, symptoms and serology, and the low level of gluten exposure (amount and duration) in this study would not be expected to cause consistent histologic damage. Others have explored the link between GIP and villous atrophy; for example, Garzón-Benavides and colleagues showed more than 4 positive urinary GIP samples out of 12 collected over a 12-month period predicted villous atrophy with 50% sensitivity and 93% specificity, and conversely, 94% of patients with negative GIP in two or more follow-up visits showed no villous atrophy [34]. Given the greater sensitivity of stool GIP testing, comparable data utilising stools are needed, and it remains to be determined if a threshold based on GIP signal and the frequency of positive tests that links to a clinically significant, unsafe level of gluten exposure can be defined.

## 5. Conclusions

The detection of GIP in stool is highly sensitive; however, it will be important to understand the significance of positive results, especially isolated elevated values in an otherwise seemingly well and adherent coeliac cohort. This includes confirming assay specificity and the factors behind inadvertent gluten ingestion. The prospective assessment of stool GIP in relevant scenarios, such as assessing patients with non-responsive or refractory coeliac disease, is an important need.

## Figures and Tables

**Table 1:** Characteristics of the study population.

	Cookie (Placebo)	Cookie (Gluten 50 mg)	Cookie (Gluten 250 mg)	Cookie (Gluten 500 mg)	Cookie (Gluten 1000 mg)	All	*p* Value
	N = 10	N = 10	N = 9	N = 9	N = 14	N = 52	
Median age (IQR), y	63 (61–66)	57 (46–65)	53 (36–60)	47 (36–50)	53 (40–62)	55 (44–62)	0.026 *
Median age at diagnosis (IQR), y	49 (46–54)	52 (33–61)	33 (24–45)	41 (30–49)	44 (28–48)	46 (32–51)	0.088
Median time on GFD (IQR), y	14 (9–17)	5 (4–9)	12 (10–20)	3 (2–6)	11 (4–14)	10 (5–14)	0.013 ^
Females, n (%)	7 (70)	7 (70)	5 (56)	5 (56)	12 (86)	36 (69)	0.498
HLA genotype, n (%)							
*DQ*2/*x*	9 (90)	8 (80)	7 (78)	(7) 78	13 (93)	44 (85)	0.387
*DQ*2/8	1 (10)	0 (0)	2 (22)	0 (0)	1 (7)	4 (8)	
*DQ*8/*x*	0 (0)	2 (20)	0 (0)	1 (11)	0 (0)	3 (6)	
* Unknown*	0 (0)	0 (0)	0 (0)	1 (11)	0 (0)	1 (2)	
Baseline assessments							
* Median symptoms* (*GSRS*)	1.3	1.1	1.6	1.5	1.4	1.3	0.270
* Median adherence* (*CDAT*)	10	9	13	10	10	10	0.618
* tTG-IgA serology* (*negative* %)	100	100	100	100	100	100	
* DGP-IgG serology* (*negative* %)	100	100	100	100	100	100	
* Urine GIP* (*negative*, %)	100	100	100	100	100	100	
* Mean bowel movements per day* (*IQR*)	1.8 (1.2–1.9)	1.4 (1.0–1.6)	1.4 (1.2–1.6)	1.4 (1.0–1.6)	1.3 (0.9–1.8)	1.5 (1.0–1.7)	0.367

Statistical analysis of groups were as follows: Chi-square to compare HLA and gender; ANOVA to compare years; Kruskal-Wallis to compare CD-GSRS and CDAT scores. * Significant difference in age between placebo and 500 mg cohorts *p* < 0.05. ^ Significant difference in time on GFD between 250 mg and 500 mg cohorts. Abbreviations: IQR, Interquartile Range; GFD, Gluten Free Diet; HLA, Human Leuocyte Antigen; GSRS, Gastrointestinal Symptom Rating Scale; CDAT, Celiac Dietary Adherance Test; GIP, Gluten Immunogenic Peptide.

## Data Availability

Data are contained within the article and supplementary materials.

## Supplementary Materials

The following supporting information can be downloaded at: https://www.mdpi.com/article/10.3390/nu16020279/s1, Figure S1: (a) Peak GIP concentration and (b) GIP AUC following known gluten exposure. Each point is representative of a single participant. Short lines represent means for each dose cohort. Statistical differences between dose cohorts were determined using Kruskal–Wallis * *p* < 0.05, ** *p* = < 0.01. Figure S2: Combined CeD-PRO symptom data for all cohorts post-gluten cookie challenge. Blue categories indicate all GI symptoms except nausea and vomiting. Orange indicates nausea and vomiting. Green indicates extraintestinal symptoms. Figure S3: GIP excretion patterns per participant during the study run-in were converted to scores based on the number of GIP-positive samples and concentration to assess correlation with the Celiac Dietary Adherence Test (CDAT) score. CONSORT 2010 checklist of information to include when reporting a randomised trial.
